# The *Biomolecules* Journal Club: Highlights on Recent Papers

**DOI:** 10.3390/biom16070973

**Published:** 2026-07-02

**Authors:** Menno Hoekstra, Natalia G. Vallianou, Iogann Tolbatov, Jiayi Dai, Chang Liu, Aejin Kim, Su-Yeon Yang, Sang-Han Lee, Anna Rita Migliaccio

**Affiliations:** 1 Division of Systems Pharmacology and Pharmacy, Leiden Academic Centre for Drug Research, Leiden University, 2333 CC Leiden, The Netherlands; 2 First Department of Internal Medicine, Sismanogleio General Hospital, 15126 Athens, Greece; 3 Department of Chemical, Physical, Mathematical and Natural Sciences, University of Sassari, Via Vienna 2, 07100 Sassari, Italy; 4 Department of Biomedical and Pharmaceutical Sciences, College of Pharmacy, University of Rhode Island, Kingston, RI 02881, USA; jiayi.dai@uri.edu; 5 School of Food Science & Biotechnology, Kyungpook National University, Daegu 41566, Republic of Korea; jinaizxen@gmail.com (A.K.); didtndus1128@knu.ac.kr (S.-Y.Y.); 6 Food and Bio-Industry Research Institute, Inner Beauty/Antiaging Center, Kyungpook National University, Daegu 41566, Republic of Korea; 7 Institute of Nanotechnology, National Research Council (Cnr-NANOTEC), c/o Campus Ecotekne, 73100 Lecce, Italy; 8 Altius Institute for Biomedical Sciences, Seattle, WA 98121, USA

## 1. What Is the Primary Source of Circulating Appetite Stimulator Acyl-Coenzyme a Binding Protein?

Highlighting the work of Meyer et al. (2025) [1], authored by Menno Hoekstra.

The circulating peptide acyl-coenzyme A binding protein/diazepam binding inhibitor (ACBP/DBI) is a potent inducer of obesity. ACBP stimulates uptake of glucose into the liver and adipose tissue while also inhibiting the loss of excess energy via fatty acid oxidation, thereby activating orexigenic neurons and increasing food intake [2]. Its obesogenic actions are thought to arise from a pathological feedforward loop in which the liver and adipose tissue primarily produce ACBP, which subsequently changes the metabolic state of these tissues. Accordingly, plasma ACBP levels are increased in obese human subjects and reduced in patients that suffer from anorexia nervosa [3]. In addition, plasma ACBP concentrations correlate strongly with the severity of metabolic dysfunction-associated steatotic liver disease [4]. However, it should be noted that the recent study by Meyer et al., published in the journal *Biomolecules,* has provided data that warrant critical reflection on this concept. More specifically, it was found that weight loss in response to bariatric surgery in morbidly obese patients was not associated with the anticipated decrease, but rather with an increase in plasma ACBP concentrations [1]. Furthermore, bariatric surgery did not significantly change ACBP transcript levels in either the hepatic or adipose tissues [1]. As such, it remains unclear what the primary source of circulating ACBP really is. Studies are thus needed in which ACBP expression is selectively eliminated from individual cell types, i.e., hepatocytes or adipocytes, to quantify the relative impact of different cell populations on plasma ACBP levels. Notably, we have found that liver Kupffer cells, rather than hepatocytes actually represent the most prominent source of hepatic and circulating cholesteryl ester transfer protein, a secreted protein that also executes a major impact on systemic lipid homeostasis [5]. As such, the possibility that macrophages are an important contributor to the total ACBP pool should not be ignored.

## 2. The Future of Chronic Kidney Disease Treatment: Combination Therapy (Polypill) or Biomarker-Guided Personalized Intervention?

Highlighting the work of Biglari et al. (2025) [6], authored by Natalia G. Vallianou.

Chronic kidney disease (CKD) is alarmingly increasing, affecting over 800 million people worldwide. The most concerning issue is that the majority of patients with CKD are unaware of their clinical condition [7].

In their article in *Biomolecules*, Biglari et al. addressed the multi-faceted burden of CKD, especially in terms of etiology, pathogenesis and treatment. Despite different etiologic factors, such as diabetes, hypertension, obesity and glomerulopathies, CKD in general has been demonstrated to share common pathogenetic pathways. Thus, the term “cardiovascular–kidney–metabolic syndrome” (CKMS) has been introduced to describe the intricate interplay among genetic factors, metabolic inflammation, fibrosis and gut dysbiosis. Regarding treatment options among patients with CKD, RAAS inhibition with angiotensin-converting enzyme (ACE) inhibitors or angiotensin II receptor blockers (ARBs) has been suggested as first-line treatment for patients with CKD and significant albuminuria according to the 2024 KDIGO Guidelines. Additionally, non-steroidal mineralocorticoid receptor antagonists (ns-MRAs), like finerenone, have been developed to overcome the adverse effect of hyperkalemia [6]. Moreover, in the FIGARO-DKD, FIDELIO-DKD and FIDELITY studies, finerenone was associated with reductions in cardiovascular (CVD) events as well as regression in GFR and albuminuria [8]. Sodium–glucose co-transporter 2 (SGLT2) inhibitors have also been documented to exert nephroprotective effects in the CREDENCE, DAPA-CKD, EMPA-KIDNEY and VERTIS-CV studies. Glucagon-like peptide 1 receptor agonists (GLP1-RAs), like liraglutide in the LEADER study and semaglutide in the SUSTAIN-6 and FLOW studies, have also shown positive kidney outcomes [9]. Endothelin receptor antagonists (ERAs), like atrasentan, reduced albuminuria in the SONAR study, while aldosterone synthase inhibitors (ASIs), like vicadrostat, may be promising for reducing CKD progression and lowering CVD events. Biglari et al. conclude that combining the aforementioned drugs in a polypill could enhance adherence to therapy and provide patients with multiple benefits. Antihypertensive effects, reductions in albuminuria, improvements in GFR and amelioration of metabolic dysfunctions are all beneficial effects of a polypill. Nevertheless, administration of a polypill requires strict monitoring to avoid adverse effects and to facilitate biomarker-guided interventions [6].

## 3. Beyond Passive Transport: How Albumin Binding Shapes the Fate of Gold and Platinum Therapeutics

Highlighting the work of Vitali et al. (2026) [10], authored by Iogann Tolbatov.

The interaction between anticancer metallodrugs and human serum albumin (HSA) has long been a subject of debate: does albumin act as a shuttle that carries the drug to the tumor or as a “sink” leading to its systemic inactivation? In a recent study published in *Inorganic Chemistry Frontiers*, Messori, Massai, Marzo, and colleagues provide a direct experimental resolution to this question [10].

Using a combination of electrospray ionization mass spectrometry (ESI-MS) and inductively coupled plasma optical emission spectroscopy (ICP-OES) to characterize HSA-bioconjugates, the authors found that biological activity varied significantly depending on the metal center. While both platinum(II) and gold(I) drugs form stable adducts with the protein, the platinum compounds (cisplatin and oxaliplatin) essentially lose their antiproliferative activity upon conjugation. The authors demonstrate that this is not merely a consequence of reduced cellular uptake; rather, the platinum–protein bond is so robust that the pharmacophore is effectively sequestered, preventing it from reaching its nuclear DNA targets.

In contrast, the auranofin–HSA conjugate retains significant cytotoxic efficacy. The study proves that the gold–albumin interaction is uniquely reversible in the presence of intracellular thiols like glutathione. This flexibility enables the gold fragment to be released from its protein carrier once inside the cancer cell, maintaining its ability to inhibit key targets such as thioredoxin reductase.

By identifying the precise chemical hurdles—irreversible sequestration versus reversible transport—this study clarifies the factors determining the success of metallodrug delivery. With a clear structural basis established, the authors offer a chemical rationale for the development of optimized albumin-based drug delivery systems in clinical oncology.

## 4. Unlocking PD-1/PD-L1 with Small Molecules: New Screening Platforms and Broader Targeting Strategies

Highlighting the works of Zamani et al. (2026) [11] and Sharma et al. (2026) [12], authored by Jiayi Dai and Chang Liu.

The PD-1/PD-L1 axis is a key immune checkpoint pathway in cancer, where PD-L1 on tumor cells binds PD-1 on T cells to suppress antitumor immunity, making it an important therapeutic target [13]. Although monoclonal antibodies have achieved major clinical success, their cost, delivery limitations, and immune-related adverse effects highlight the need for alternative modalities such as small-molecule inhibitors, which remain less developed [13,14]. Recent studies in *ACS Omega* and *PLoS ONE* describe complementary experimental and computational strategies to accelerate discovery in this area.

Mohammad et al. developed the DNA-linked Inhibitor Antibody Assay (DIANA), a multiwell, plate-based sandwich-format platform for high-throughput screening of PD-1/PD-L1 inhibitors [11]. In this assay, NeutrAvidin-coated plates capture biotinylated anti-His iBodies bound to recombinant human PD-L1, and binding events are quantified by qPCR. DIANA demonstrated strong performance, including throughput of tens of thousands of compounds per day, tolerance of up to 10% DMSO, and excellent robustness (Z′-factor = 0.94). Screening >6400 compounds from in-house and α-helix peptidomimetic libraries yielded 24 initial hits; however, these were later identified as false positives in cellular PD-1/PD-L1 assays, suggesting that larger or more diverse libraries may be required. Nevertheless, DIANA represents a valuable screening platform for future inhibitor discovery.

Neha et al. applied an integrated computational workflow to screen 17,967 phytochemicals from 4010 Indian medicinal plants against PD-1 [12]. Following structure-based virtual screening and consensus molecular docking using seven algorithms, promising compounds were further evaluated by density functional theory, 300 ns molecular dynamics simulations, and ADME profiling. Among the 31 shortlisted compounds, IMPHY004834 (Mahuannin D) showed the most stable interactions and favorable pharmacokinetic properties. However, low GI absorption, poor solubility, and predicted CYP inhibition suggest that parenteral administration may be preferable.

Together, these studies highlight how complementary experimental and in silico screening approaches can accelerate the development of small-molecule PD-1/PD-L1 inhibitors.

## 5. Autophagy and Melanophagy in Skin Aging: Convergence with Epigenetic Regulation

Highlighting the work of Lee et al. (2024) [15], authored by Aejin Kim, Su-Yeon Yang, and Sang-Han Lee.

Autophagy is a central cellular quality control mechanism that maintains proteostasis and safeguards cellular homeostasis under stress [16]. Its functional decline is a defining feature of aging and contributes to the progressive accumulation of damaged proteins and organelles in skin tissue. Recent studies have further implicated selective autophagy pathways, particularly melanophagy, in the regulation of pigmentation dynamics. In this context, melanosomes are actively cleared through ubiquitin-dependent, lysosome-mediated pathways, indicating that degradation processes play a central role in maintaining pigment homeostasis. Mechanistic studies have also begun to clarify the molecular basis of melanophagy, identifying autophagy receptors such as OPTN and ubiquitin-linked signaling pathways involved in selective melanosome clearance [15]. These findings suggest that melanophagy may contribute to melanocyte adaptation under oxidative and ultraviolet stress conditions associated with skin aging.

From a translational perspective, dietary bioactive compounds, including epigallocatechin gallate and resveratrol, converge on the AMPK–mTOR signaling axis to modulate autophagic activity [17]. However, it remains unclear whether these compounds selectively regulate melanophagy rather than autophagy more broadly, and this distinction warrants further investigation. Beyond metabolic regulation, recent advances in epigenetic rejuvenation further challenge the view of aging as irreversible. Partial reprogramming studies suggest that age-associated loss of epigenetic information can be modulated [18]. Together, these observations indicate that metabolic regulation, organelle turnover, and epigenetic plasticity are closely interconnected in maintaining cellular resilience and pigment homeostasis during aging. Collectively, current findings suggest that melanophagy may represent an important yet insufficiently understood regulatory process in skin aging biology. Further studies integrating time-resolved analyses and systems-level approaches will likely be important for clarifying how turnover-associated pathways contribute to tissue aging and pigmentation dynamics.

## 6. Clonal Hematopoiesis of Indetermined Potential Meets CAR T Therapy

Highlighting the work of Saini et al. (2022) [19], authored by Anna Rita Migliaccio.

The field of clonal hematopoiesis and its implications for cancer started in 1976 with the paper by Adamson et al. [20], which exploited the recently discovered lyonization process of the X chromosome, generating two distinguishable hematopoietic stem cell (HSC) clones in females, to demonstrate that the red cells of female patients affected by Polycythemia Vera derive from one HSC clone. Advances in our understanding of the epigenetic modifications occurring during ontogenesis led us realize that, at birth, HSC form a pool of clones that are genetically identical but epigenetically different. Genetic tracking of individual HSC clones subsequently indicated that, in adults, a limited number of HSCs are active at any given time and that hematopoiesis eventually becomes monoclonal with aging [21]. When the prevailing HSC clone acquires mutations in selected genes, most commonly genes encoding epigenetic modifiers, it gives rise to clonal hematopoiesis of indeterminate potential (CHIP), which is a benign condition per se but renders HSC susceptible to acquiring additional mutations, increasing the risks of developing leukemia [22]. In addition, CHIP deeply impairs the innate immune response, increasing the risk of developing a wide range of immune-related disorders (from Alzheimer’s disease to cardiovascular disorders) and increasing the severity of manifestations associated with solid cancers [23].

CAR T represents a new frontier for cancer immunotherapy, and its clinical applications are expanding so rapidly that reviews, perspectives and research articles dedicated to this subject monopolize issues of journals dedicated to the field of cell therapy [24]. However, CAR T therapies are also associated with side effects that can be severe enough to become fatal in some patients. Saini et al. [19] reported that the presence of CHIP correlates with the severity of the cytokine storm and neurotoxicity induced by CAR T therapy in a small cohort of large B-cell lymphoma patients. Mechanistically, this correlation is somewhat expected given the numerous indications that CHIP increases the severity of immune reactions [24]. Although the patient cohort analyzed in this retrospective study was small, and the authors disclosed a mismatch between the DNA sequencing data and the precise identity of the patients [19], this paper is important because it provides testable hypotheses that may help prioritize and stratify patients for CAR T therapy. These findings warrant confirmation in larger, possibly prospective studies involving patients undergoing CAR T therapy for multiple cancer types.
